# Hierarchical Agglomerative Clustering of Bicycle Sharing Stations Based on Ultra-Light Edge Computing

**DOI:** 10.3390/s20123550

**Published:** 2020-06-23

**Authors:** Juan José Vinagre Díaz, Rubén Fernández Pozo, Ana Belén Rodríguez González, Mark R. Wilby, Carmen Sánchez Ávila

**Affiliations:** Group Biometry, Biosignals, Security, and Smart Mobility, Departamento de Matemática Aplicada a las Tecnologías de la Información y las Comunicaciones, Escuela Técnica Superior de Ingenieros de Telecomunicación, Universidad Politécnica de Madrid, Avenida Complutense 30, 28040 Madrid, Spain; ruben.fernandez@upm.es (R.F.P.); abrodriguez@etsit.upm.es (A.B.R.G.); mrwilby@etsit.upm.es (M.R.W.); carmen.sanchez.avila@upm.es (C.S.Á.)

**Keywords:** bicycle sharing systems, hierarchical clustering, edge computing, docking stations, spatio-temporal profiling, Internet of shared bicycles

## Abstract

Bicycle sharing systems (BSSs) have established a new shared-economy mobility model. After a rapid growth they are evolving into a fully-functional mobile sensor platform for cities. The viability of BSSs is floored by their operational costs, mainly due to rebalancing operations. Rebalancing implies transporting bicycles to and from docking stations in order to guarantee the service. Rebalancing performs clustering to group docking stations by behaviour and proximity. In this paper we propose a Hierarchical Agglomerative Clustering based on an Ultra-Light Edge Computing Algorithm (HAC-ULECA). We eliminate the proximity and let Hierarchical Agglomerative Clustering (HAC) focus on behaviour. Behaviour is represented by ULECA as an activity profile based on the net flow of arrivals and departures in a docking station. This drastically reduces the computing requirements which allows ULECA to run as an edge computing functionality embedded into the physical layer of the Internet of Shared Bikes (IoSB) architecture. We have applied HAC-ULECA to real data from BiciMAD, the public BSS in Madrid (Spain). Our results, presented as dendograms, graphs, geographical maps, and colour maps, show that HAC-ULECA is capable of separating behaviour profiles related to business and residential areas and extracting meaningful spatio-temporal information about the BSS and the city’s mobility.

## 1. Introduction

Bicycle sharing systems (BSSs) have emerged as a sustainable, healthy, and environmentally friendly transport mode. Its cooperative nature adds a novelty factor that follows the new economy paradigm that many cities worldwide are establishing in every domain, transport included. At present, we can find more than 500 BSSs around the world [1]. This evident success has also extended to the academic field, where the number of scientific papers has rapidly increased in the past 5 years [2].

At first, these scientific works focused on solving the basic problem BSSs face: the design of the network of bicycle stations, which is key to the eventual success of the system [3]. This factor is specially relevant given that, in most cases, BSSs are funded by public administrations, which are always restricted to fixed budgets and thus need to maximize their investment [4]. This is yet an unresolved problem that requires extended efforts from both scientific and technological fields.

Furthermore, an optimally designed and deployed BSS may still not be viable as it has to face a fundamental issue: its operational costs. Every BSS presents an unbalanced demand on the stations within its network. In addition, this disproportion occurs in both space and time, which increases the complexity of the problem. In order to solve it, BSSs perform *rebalancing* operations. The fundamentals of rebalancing are simple: a truck distributes bicycles among the set of stations in order to guarantee that both bicycles and docks are always available throughout the day. The truck can either get bicycles from a central depot or move them from one station to another. Consequently, rebalancing is essentially a routing problem that can be mathematically formulated [5].

In order to solve it in an efficient way, rebalancing must include a first phase of clustering, which groups the docking stations into sets with similar characteristics. Clustering can dramatically reduce the total length of the routes trucks have to travel to move bicycles to and from docking stations. This has a major positive impact on the operational costs and on the reduction of the associated emissions. However, the real impact of clustering greatly extends beyond rebalancing as it provides a meaningful knowledge about the actual operation of the BSS and the mobility in the city.

Once we overcome these issues, a BSS becomes not only an interesting transport mode that provides health and environmental benefits [6], but also a potential platform to collect relevant data from the city. Under this perspective, shared bicycles evolve into mobile sensors, able to retrieve quality air measurements, noise levels, or congestions, among others [7]. Thus, BSSs develop into narrow-band Internet of Things (NB-IoT) applications [8]. This transition opens up a new set of challenges. At a local level, we need to reduce energy [9] and computation costs by means of data-fusion [10] or smart routing [11]. At a global level, the BSS needs to adopt new models in order to deal with the issues this extended complexity imposes. In this respect, a BSS have been recently approached as the Internet of Shared Bikes (IoSB) [12].

The IoSB model allows us to separate the different layers we need to provide the whole set of services associated with BSSs. In addition, it contributes to the optimization of the efficiency of the computation and communication processes. In this environment, edge computing emerges as a means to optimize the use and operation of each docking station [13].

In the present work, we focus on the clustering of docking stations of a BSS, adopting the IoSB model. Consequently, we first introduce edge computing into the layered architecture of an IoSB. In order to guarantee the viability of the edge computing scheme, we reduced to the minimum the calculation requirements. As a result, we created an ultra-light edge computing algorithm (ULECA), which fully characterizes the operation of each docking station.

The output of the ULECA then feeds a hierarchical agglomerative clustering (HAC) that groups stations considering their behaviour pattern alone, thus not including any other prefixed variable like geographical proximity as in rebalancing. Therefore, HAC contributes to both the reduction of operational and environmental costs and the extraction of meaningful knowledge about mobility profiles within the BSS.

We applied this technology to real data collected from BiciMAD, the public BSS managed by Empresa Municipal de Transportes de Madrid (EMT), which belongs to the municipality of Madrid (Spain). The observed results show the capability of the HAC-ULECA to perform an intelligent clustering of docking stations that detects spatio-temporal correlations, inherently related to geographical proximity and the main use of the area where they are located (residential, business, commercial, transport, education, etc.). Consequently, HAC-ULECA provides key information to the BSS managers in order to (i) redesign or extend the existing network based on the knowledge about the city’s behaviour; (ii) construct accurate predictions of the expected demand to optimize the operation of the system; and (iii) design high-performance dynamic rebalancing schemes to reduce the costs and improve the perceived quality of the BSS.

## 2. Related Work

Given that the fundamental operational cost of a BSS is rebalancing, most scientific works related to clustering simply take it as a first step in the process. Accordingly, clustering aims at characterizing BSS docking stations in terms of behaviour patterns in order to predict their future demand. This prognosis will then feed a dynamic rebalancing algorithm to be triggered whenever it is required. Rebalancing is typically static in current BSSs, which is evidently inefficient.

In order to overcome this issue, rebalancing processes seek to minimize three variables [14]: (i) the fraction of time the stations have no available bicycles or docks; (ii) the number of rebalancing operations; and (iii) the travelled distance. Considering these three objectives, rebalancing can be formalized as a dynamic resource redistribution based on demand estimation in the field of BSSs [15]. Under this perspective, scientific activity focuses on designing rebalancing algorithms, which commonly include three phases [16]: (i) clustering; (ii) prediction; (iii) and intra-cluster and inter-cluster routing.

This approach to clustering directs its particular goals to minimizing the three variables that impact on the performance of rebalancing. This leads to imposing two criteria to the grouping of docking stations: geographical proximity and similarity on their behaviour. The former reduces the length of the rebalancing route; the latter is required to anticipate the need of bicycles or docks and trigger a rebalancing operation. However, we could alleviate this imposed requirements as behaviour itself may embed geographical information.

Clustering is a complex task that entails a set of challenges [17]. First, it is affected by multiple factors like time of day, meteorology, and special events like concerts or protests, among others. Second, these factors are unbalanced, which complicates the generalized characterization of the behaviour of docking stations. Third, the trips the users perform usually show a high variability, which complicates the task of finding similarities between the docking station’s behaviour. In order to tackle these challenges, the scientific community has proposed a diverse set of techniques, which indicates that there is no consensus about the optimal solution to this problem and it is still open to further research.

The most basic approach to clustering exclusively considers the location of the docking stations [18]. This avoids the complexity of dealing with behavioural information, but also considerably limits the obtained knowledge about the BSS. A first category that includes both factors proposes expectation-maximization (EM) algorithms as the clustering technique. This requires the calculation of a-posteriori probabilities, which is not always viable given the available data. Among this category, several works studied behavioural patterns in cities like Barcelona (Spain) [19] and Paris (France) [20] in order to discover temporal and spatial patterns of human behaviour, easily linked to day of week and specific facilities like parks and train stations or type of area (residential, business, etc.).

A second category of works uses *k*-means as the core of their clustering algorithm [17]. In this case, a third factor is considered: similarity between origin–destination transitions among groups. The proposed model was applied to real-world datasets from New York and Washington D.C. This approach may fail in situations where clusters are non-convex and there are physical barriers such as rivers. For this reason, the authors in [21] processed data collected from the New York City Bike System and proposed a spectral clustering algorithm that operates in conjunction with a label propagation algorithm, which controls the geographical extension of the obtained clusters. According to the obtained results, it seems that imposing geographical proximity as a requirement for clustering somehow fades the information regarding the actual behaviour shown at docking stations.

A third category incorporates labelled data that leads to supervised clustering algorithms [22]. The labels are based on temporal information (time of day and day of week) and allow a joint maximization of purity and similarity in each cluster. This approach was applied to a BSS in the San Francisco Bay Area in order to detect unbalanced situations. The presented results show its scalability, speed, and simplicity. Nevertheless, the algorithm shows a significant drawback: it only factors in the present information about the availability (ratio between the number of available bikes and the total capacity) of each docking station, which does not take into account whether the bicycles were picked up or returned by users or rebalancing operations. This same disadvantage is observed in [16].

Anyhow, the idea of incorporating temporal information to the algorithm is clearly beneficial for clustering given that there is an obvious relation between a docking station’s behaviour and the time of day or the day of week. The authors in [23] use this relation to construct a Graph Convolutional Neural Network in order to detect complex connections among the behavioural patterns observed in different docking stations. They describe the clustering problem as a non-Euclidean space, which consequently requires specific distance matrices. This particular need significantly increases the complexity of solutions and its subsequent computing load.

This evident complexity inherent to clustering of docking stations in BSSs has redirected scientific literature towards adopting an IoSB model [12]. Under this perspective a BSS comprises five layers: perception, physical, communications, application, and security. Among them, the physical layer is responsible for the system management, which includes the interaction with the user during the process of picking up and returning the bicycle. The physical layer could be extended to perform the characterization of the docking station, using an edge computing approach. This way, we could move this calculation to the edge, alleviating the data load on the communications channel to the central or cloud server. Following this approach, the authors in [1] designed an IoSB architecture for a BSS system, which includes three basic layers: the site, the BSS server, and the BSS cloud centre. They proposed a clustering algorithm based on self-organizing regression to be performed in the edge (site). However, this method may imply high computational requirements, exceeding the capacity of the site. In this case, the edge will require to offload this calculation to the upper BSS server layer.

In view of these previous works, this paper proposes a novel approach to the clustering of docking stations in BSSs. First, we adopt the IoB architecture, inserting edge computing functionalities into the Physical layer. The calculations to be performed at the edge must be light enough in order to guarantee the viability of this proposition. Consequently, we have designed the ULECA, which ensures its operation in the docking station site, using the already existing infrastructure. In addition, we adopt an innovative perspective, treating clustering as a separate problem, with no prior connection to rebalancing. This allows us to build a clustering functionality that generates significant information for different applications in the upper layers of the IoSB, ranging from demand prediction to global knowledge about the city’s mobility. Separating clustering from rebalancing also let us avoid imposing geographical proximity factors to clustering, which is only relevant as a rebalancing requirement. Our proposed HAC-ULECA approach forms clusters based exclusively on the similarities in the behaviours shown by docking stations. As an illustrative example, the docking stations close to two football stadiums may exhibit identical patterns despite the distance between them. Our results show that the behaviour observed in docking stations somehow carry geographical information.

## 3. Hierarchical Agglomerative Clustering Based on Ultra-Light Edge Computing Algorithm

### 3.1. IoSB Architecture

Following the approach previously described, in the current section we describe the HAC-ULECA. This algorithm provides a highly reliable clustering method with very low computational needs. The key to achieve this purpose is to treat each docking station individually, thus avoiding modelling how demands are interrelated among them. In addition, we separate clustering from rebalancing processes, which removes the imposed geographical proximity requirement, thus alleviating the computational load. As an additional positive consequence, considering clustering as a separate process arises the intrinsic similarities in behaviour among the docking stations, which are no longer biased by their location.

Furthermore, this approach eliminates the need to build a graph of the BSS, which also inserts extra complexity to the problem. Under our perspective, a docking station is represented as an object into the IoSB hierarchy [12], with functionalities within the perception and physical layers, which uses the communication layer to transmit the relevant information to the upper processing levels. This approach also matches the edge-computing architecture proposed in [1], where the BSS is decomposed into site, BSS Server, and BSS cloud center.

We merge these two complementary models and build an overall process that entails a set of steps. First, we take as our basic information, the number of arrivals and departures from the docking station. These data is provided by the sensors installed in each dock, forming the perception layer on the site. Without leaving the site, its physical layer receives these data and the ULECA builds a feature vector of the behaviour of the docking station in each time slot. This information is then handed to an upper processing layer that resides in the BSS Server, able to connect to the different sites of the BSS. At this point, we run the HAC in order to measure similarities among each pair of objects in a sequence of steps that starts with docking stations and evolves to clusters. Finally, the results we obtained are visualized using dendograms and geographical information systems in order to provide meaningful information and extract conclusions.

### 3.2. Problem Statement

We can state the clustering problem as follows. Consider a BSS represented by the set S consisting of *S* docking stations. For each docking station we know the number of arrivals and departures at prefixed time intervals. These time intervals are determined by the BSS itself, which provides aggregated data for operational and privacy issues, thus generating data at t=1,2,…,T, with *T* the total number of time intervals in the dataset. Let $A_{i}\left(t\right)$ be the number of bicycles that are returned to a docking station *i* during the time period *t*, and let $D_{i}\left(t\right)$ be the number of bicycles that are picked up at the same docking station in that same period of time. Both $A_{i}\left(t\right)$ and $D_{i}\left(t\right)$ are independent of the state of docking station *i* at instant *t*, except the obvious situation of having no available bicycles or docks [24].

Thus, we aim at creating *K* clusters of docking stations, each of which with a variable number of elements $n_{i}$. These clusters must share a common feature vector related to their behavioural pattern throughout a certain period of time, *T*. We will refer to this behavioural pattern as the *activity profile*$a_{i}\left(t\right)$.

### 3.3. Ultra-Light Edge Computing Algorithm

The ULECA is fed with the data provided by the sensors in the docks that measure the number of arrivals $A_{i}\left(t\right)$ and departures $D_{i}\left(t\right)$ at time *t* to generate the activity profile $a_{i}\left(t\right)$. We define the activity profile in station *i* as:

$$a_{i}\left(t\right)=\mathrm{sgn}\left(A_{i}\left(t\right)-D_{i}\left(t\right)\right),$$

where sgn(·) denotes the *signum* function.

Note that this activity profile can only take values from the set −1,0,1 where:

−1: indicates a negative net arrival, i.e., the number of bicycles that have been returned is lower than the number of bicycles that have been picked up; in this case, the docking station is empting;
+0: indicates that the number of deposits compensate the number of withdrawals, thus resulting in a constant occupancy value during time interval *t*;
+1: indicates a positive net arrival, i.e., the number of bicycles that have been returned is higher than the number of bicycles that have been picked up; in this case, the docking station is filling up.

The ULECA focuses on the net flow observed in each docking station during each time interval. This way it avoids any dependency on the capacity of the docking stations thus reflecting specifically their behaviour rather than their ability to guarantee available bicycles and docks through time as rebalancing requires. The approach we adopt takes the same basis as in [14], where the authors characterize a docking station’s behaviour with a birth-death model that matches our arrival-departure measurements. However, they then try to model the interdependency of the demands among docking stations by means of a Markov process. As we described in Section 2, many previous works follow this same route, aiming at modelling the joint demand of each pair of docking stations within the BSS network. This leads to a demand matrix [23] where they have to calculate every aggregated demand between each pair of docking stations *i* and *j*, in both directions. In addition, they have to calculate a demand correlation matrix using some correlation measurement like the Pearson correlation coefficient. The fundamental problem to solve is then to find the adjacent matrix, which significantly increases the complexity of the solution and makes it inviable from an edge computing perspective. This process can be substantially simplified by analysing each docking station in isolation. This way we can analyse its particular behaviour and rely on an intelligent clustering technique to deal with the interrelation issue.

### 3.4. Hierarchical Agglomerative Clustering

The activity profile obtained from the ULECA feeds a second processing level running the HAC. HAC groups docking stations based on their similarity in their behaviour throughout time.

In order to do so, we define a non-Euclidean distance, that represents the correlation between two given docking stations. In this respect, we propose the following semimetric that provides a numerical value representing the disparity between each pair of activity profiles along the predefined time interval *T*:

(1) $$d\left(i,j\right)=\frac{1}{T}\cdot \left(T-\sum_{t=1}^{T}\left[a_{i}\left(t\right)=a_{j}\left(t\right)\right]\right).$$

In Equation (1), we use the Iverson bracket notation ($\left[\cdot \right]$) that generalises the Kronecker delta.

Note that the function defined in Equation (1) calculates the value of d(·), with 0≤d(·)≤1, which is not a distance itself given that, although it meets the first three axioms (non-negativity, identity of indiscernibles, and symmetry), it does not necessarily satisfy the triangle inequality.

From Equation (1) we can derive a similarity index

(2) s(i,j)=1−d(i,j),

which admits a straightforward interpretation: the similarity between two docking stations measures the percentage of time *T* in which both showed identical qualitative behaviour regarding the flow of bicycles.

Based on the established semimetric between two specific elements (docking stations) in the set S, we then link different groups of docking stations imposing certain linkage criterion. To this purpose, we define the distance between clusters *X* and *Y* as:

(3) $$D\left(X,Y\right)=\min_{i\in X,\;j\in Y}d\left(i,j\right).$$

The overall process that HAC-ULECA implements is next described. We start by calculating the value of d(i,j) for every pair of docking stations within the BSS taken just once, given that d(i,j) satisfies the symmetry axiom; this results in S(S−1)/2 values.

Next we sort the docking stations given these values. In order to do this, we first assign the two stations with the lowest d(i,j) to the first two positions in the list. Then we take the next value of d(i,j) and place the corresponding docking stations in the next two places, if they had not already been given a position in the list. We repeat this process along the complete set of values of d(i,j). At this point, we have obtained an ordered list of docking stations depending on their similarity with at least another one in the BSS.

Then, we create *S* clusters containing a single docking station following the corresponding ordered list. At this moment we proceed to perform the hierarchical agglomerative routine: we start by creating the union of the two first clusters; next we run a loop that takes the next single-station cluster $K_{i}$ in the list, calculates $D(K_{i},K_{j})$ being $K_{j}$ any other already built cluster in order to find the cluster containing the docking station that minimizes d(i,j), and perform the union between these two, i.e, $K_{i}\cup K_{j}$. At the end of this first round, every docking station in the network has been assigned to one of the *n* resulting clusters.

Finally, we repeat this same process going through the set of clusters. In each step of the loop, we take one of the *n* clusters and join it with the one in the set that minimizes D(·). This iterative process ends when the algorithm forms 1 cluster containing a hierarchy of all the docking stations in the BSS. We can now obtain whichever number of clusters just by going back through this hierarchy and stopping when we reach the desired number of clusters.

## 4. Results

In order to test the performance of the proposed methodology, we applied the HAC-ULECA to a real BSS in the city of Madrid (Spain). In this section we present the results we have obtained. Looking for a means to show the potential of HAC-ULECA to extract knowledge about the similar behaviour of docking stations within clusters, we provide four ways of visualizing the results: dendograms, graphs, geographical maps, and temporal colour maps.

### 4.1. Dataset

We used data from BiciMAD, a public BSS managed by the Empresa Municipal de Transportes (EMT) within the Municipality of Madrid (Spain). BiciMAD includes a total of 172 docking stations (4095 docks), 169 of which are currently active (4023 docks).

The data we used corresponded to a complete month (February, 2019). Each element in the dataset reflects a single trip, from an origin to a destination. The dataset included information about 303,962 trips. The data associated to each of these trips included:

- Time stamp: it showed the moment when the bicycle was picked up; every time stamp was set with 1 hour definition for privacy and anonymity issues.
- User’s identifier: it was an encrypted identifier which was unique per user and day in order to preserve the privacy of the user.
- Type of user: annual, eventual, staff.
- User’s range of age: it provided six age intervals [0,16], [17,18], [19–26], [27–40], [41–65], [66,∞), and unknown.
- Identifier of the origin docking station.
- Identifier of the destination docking station.
- Travel time: time elapsed between the withdrawal and deposit of the bicycle.
- Track: sequence of geographical coordinates travelled by the bicycle, updated every minute (this information was not always available).

Among the features these data carried, the time stamp was specially relevant in the calculation of the similarity index s(i,j) defined in Equation (2). From a sensitivity analysis point of view, the similarity index was affected by the time interval Δt. Very low values of Δt may have induced low similarity indexes for a given pair of docking stations, which did not reflect their actual comparative behaviour. On the contrary, large values of Δt may have shown just an average similarity, hiding the specific features of the behaviour of docking stations, which may have been required to classify them correctly. In our case, BiciMAD recorded data in 1-hour intervals, which was a sensible value for Δt.

### 4.2. Dendograms

**Figure 1:**
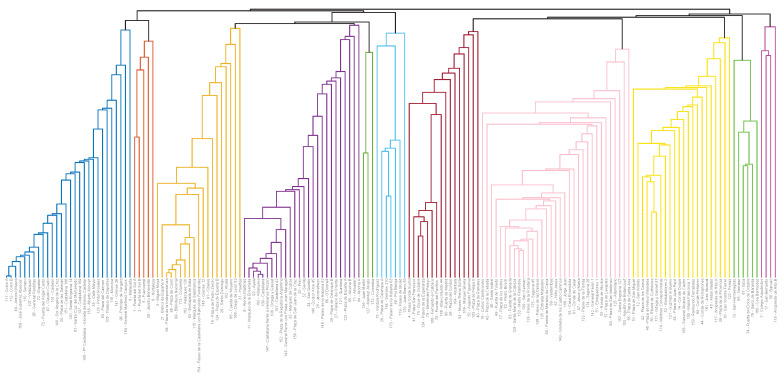
Dendrogram (binary tree). Groups numbered from left to right: g#1 (dark blue), 28 stations; g#2 (red), 5 stations; g#3 (orange), 19 stations; g#4 (purple), 26 stations; g#5 (dark green), 3 stations; g#6 (light blue), 7 stations; g#7 (garnet), 16 stations; g#8 (light pink), 33 stations; g#9 (gold yellow), 22 stations; g#10 (light green), 6 stations; g#11 (dark pink), 4 stations.

The dendogram is a useful tool to show how the HAC methodology operates. It shows a tree-structure of a hierarchical network starting from a root node that is then sequentially split until it reaches the leaves. Applying this visualization technique to the results we obtained from the HAC-ULECA we generated the dendogram in Figure 1.

The bottom of the dendogram shows all 169 docking stations. For clarity purposes, we depict them in the order that best fits the visual interpretation of the dendogram; thus note that this order does not follow the sorting by similarity described in Section 3.4.

This representation allows us to observe that HAC recursively attached docking stations to already existing clusters creating a hierarchical binary tree. The HAC algorithm was first applied to the original 169 single-station clusters. At the end of this phase, HAC built 11 clusters. We assigned a specific name (g#1–g#11) and colour to these clusters, which we will use throughout the remainder of this paper.

Then HAC repeated the same process over this set of 11 clusters. Every step of this iteration joined one cluster to some other among the already existing, applying the same distance criterion. The links it created are shown as black lines that connect different clusters, at the top part of Figure 1. This process ended when HAC-ULECA reached a single cluster containing the overall hierarchy.

### 4.3. Analysis with Two Clusters

Once we have built the hierarchy provided by HAC-ULECA, we can choose whichever number of clusters we want, to perform specific analysis on the BSS. Let us start from the simplest one in order to first highlight some features in the performance of the methodology and then extract meaningful information about the behaviour of BiciMAD.

Taking the first 2 levels of the hierarchy results in clusters *A* with 88 and *B* with 81 docking stations. Cluster *A* was formed by the union of first level clusters g#1 to g#6 in Figure 1. Accordingly, cluster *B* was formed by the union of first level clusters g#7 to g#11.

**Figure 2:**
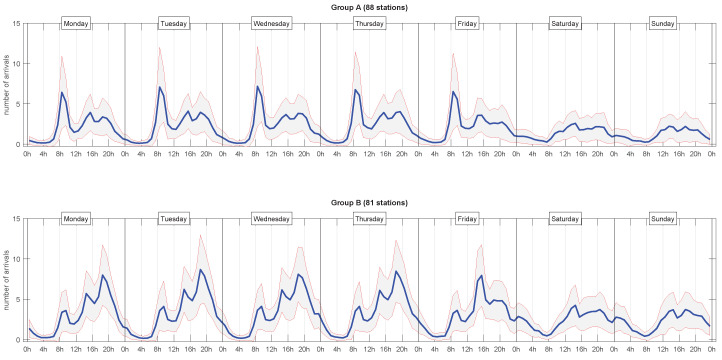
Weekly arrival profiles in clusters *A* and *B*. Mean plus/minus a standard deviation.

Figure 2 shows the arrival profiles shown at both clusters, separated by day of week. We can clearly observe that, in working days, cluster *A* was characterized by a peak in the morning, whilst cluster *B* shows it in the afternoon. Analysed as whole, these two clusters reflected a typical commuter mobility scheme, where the arrival profiles of clusters *A* and *B* corresponded to business and residential areas.

**Figure 3:**
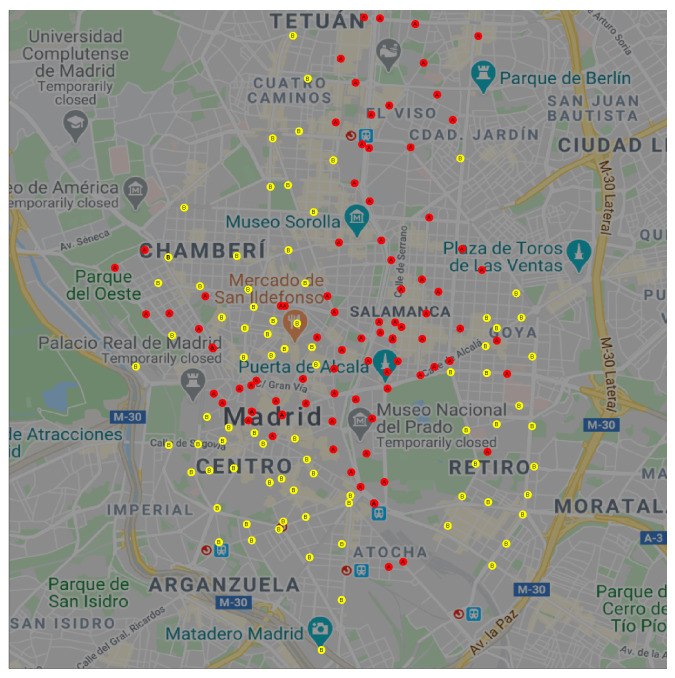
Spatial location of docking stations in clusters *A* (red) and *B* (yellow).

We can extend this observation to a spatial visualization on a map. Figure 3 shows the geographical location of each docking station in BiciMAD; clusters *A* and *B* are represented in red and yellow circles respectively.

We can observe that docking stations in cluster *A* were located in Madrid’s down-town, the business area at the North-East, the main South-North and East-West arterials, and near the primary transport facilities (train and bus stations). On the other hand, docking stations in cluster *B* were located in Madrid’s residential areas that lay on the periphery of the city.

**Figure 4:**
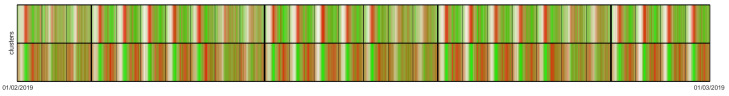
Colour map for 2 clusters.

In addition, we developed a new way of visualizing these data. The colour map in Figure 4 shows an integrated activity profile of each cluster (*Y* axis) in each 1 hour time interval in the dataset (*X* axis). Docking stations in clusters *A* and *B* were grouped at the top and bottom parts of the map, separated by a horizontal line. We also incorporated thin vertical lines for each day and thick vertical lines for each week. For each docking station and hour, we assigned red to positive, green to negative, and white to zero net arrivals. Then we merged these into a common overall weighted colour that represents the integrated activity profile of each cluster (*A* and *B*) at a given hour.

This visual representation of the overall behaviour of each cluster resulting from HAC-ULECA allows us to observe that there were actually two separate patterns. Cluster *A* showed a white-red-green pattern during working days, which we could easily associate with business areas where users start arriving in the morning (red band on the left of each working day) and departing in the afternoon (green band on the right of each working day). On the contrary, cluster *B* was characterized by a white-green-red pattern that corresponded to residential area for analogous reasons. We can also notice that white bands were broader in cluster *B* than in cluster *A*. This meant that the inactivity periods (mostly night hours) were shorter in residential areas than in business areas as we expected to happen.

### 4.4. Analysis with 11 Clusters

Let us now analyse the results observing the 11 clusters (g#1 to g#11) HAC-ULECA built in its first round.

**Figure 5:**
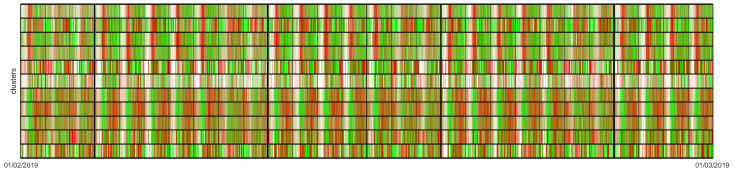
Colour map for 11 clusters.

Figure 5 shows the colour map of the activity profiles detected in theses clusters, placed in ascending order from g#1 at the top to g#11 at the bottom. As we can observe, clusters at the top part of the colour map mainly presenedt a profile of the type white-red-green, whilst clusters at the bottom part of the colour map matched the profile white-green-red. This observation fits with what we concluded in Section 4.3: HAC-ULECA detects business and residential area profiles.

Let us extend this analysis within these two primary sets of clusters. Among the clusters that formed these two basic profiles, we found some with a stable pattern. This is the case of clusters g#1, g#3, and g#, within the business area profile, and clusters g#7, g#8, and g#9 within the residential area profile. The rest (g#2, g#5, g#6, g#10, and g#11) presented a somehow noisy behaviour. Among this last set, cluster g#6 showed a special patter, characterized by a predominant white colour that could not be observed among the rest. We next proceeded to study the reasons for these different behaviours.

**Figure 6:**
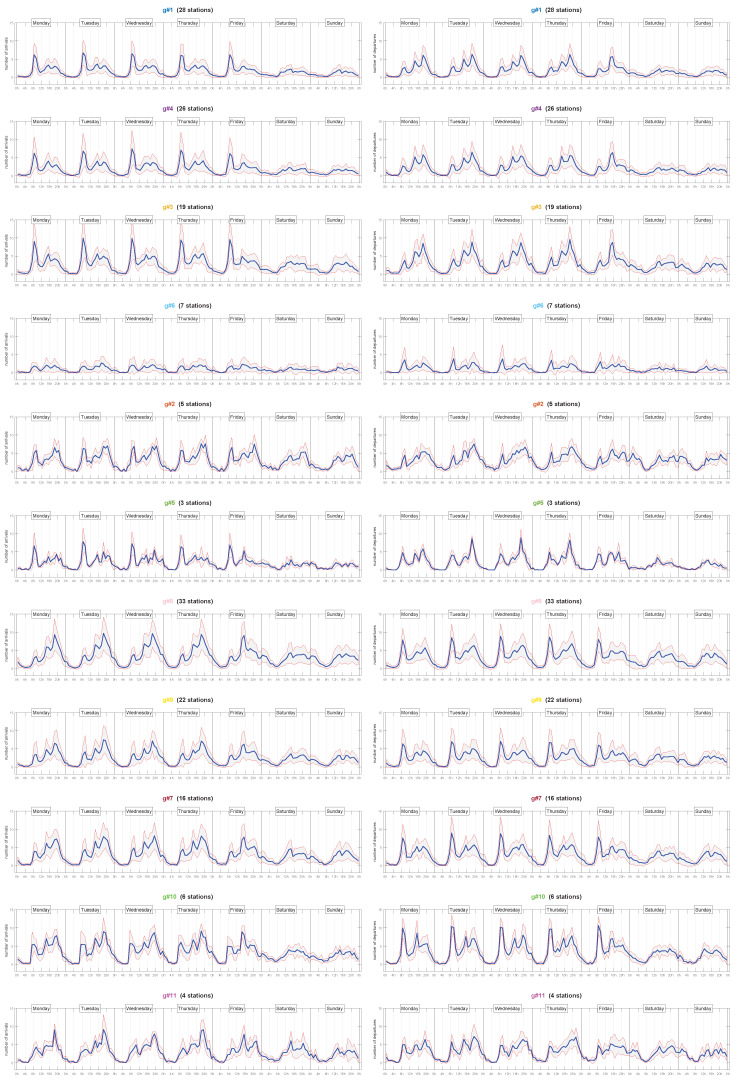
Weekly profiles for 11 clusters: arrivals (left); departures (right).

Figure 6 shows the arrival and departure profiles of each cluster and day of week. We grouped separately clusters with business and residential area profiles. Within each group, we ordered the graphs from top to bottom, according to the number of stations in the cluster.

We can observe that the stability of the pattern in a cluster that we saw in Figure 5 was related to the number of docking stations it includes. Thus, the bottom-most clusters coincided with noisy patterns that did not exactly match those exhibited by the clusters at the top. A detailed study on the dendogram in Figure 1 reveals that these noisy clusters were just linked to the rest at the upper hierarchical layers, where HAC-ULECA joined the clusters with the lowest levels of similarity to the already built.

In addition, cluster g#6 had its own particularity as it grouped the docking stations that presented failures throughout the month under study. This was the reason for having such a white-like behaviour.

**Figure 7:**
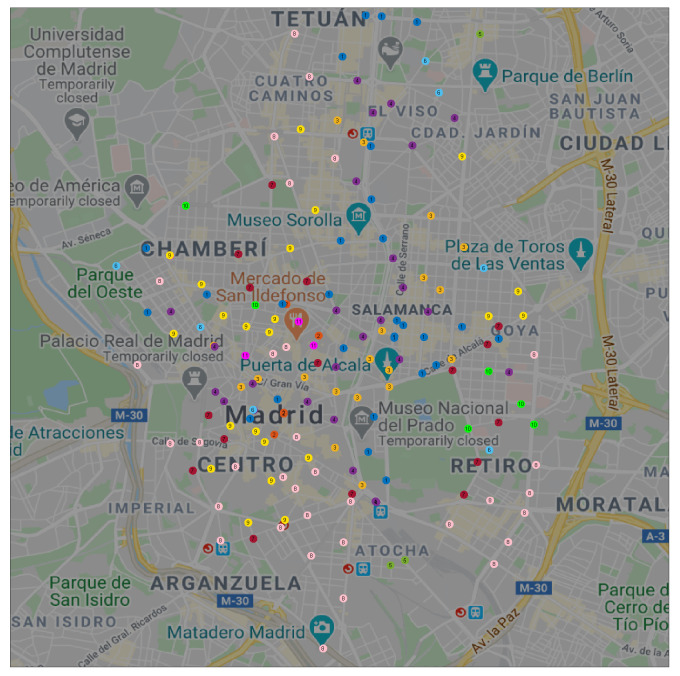
Spatial location of stations for 11 clusters.

Figure 7 shows these 11 clusters on a geographical map. We can observe that clusters g#1, g#4, and g#3 covered business areas. Docking stations in cluster g#2 were always close to some other in cluster g#1; HAC-ULECA detected this relation and merged these two clusters in the last steps of the process. Cluster g#5 was only formed by 3 docking stations and was incorporated to the business area profile the latest.

On the other hand, clusters g#8, g#9, and g#7 covered residential areas of the city. Once again, the clusters with a low number of docking stations (g#10 and g#11) were linked to these first three on the last steps of the algorithm.

Finally, we can observe that cluster g#6 was formed by a scattered set of docking stations. This fact suggests, as expected, that failures were not condensed into a specific area in the city.

## 5. Conclusions and Future Research

We have proposed HAC-ULECA as a clustering methodology for BSSs that follows the IoSB model in [12] and inserts edge computing functionalities into its physical layer. Docking stations are characterized considering their behavioural patterns throughout time. In order to guarantee the viability of an edge computing approach, the ULECA calculates activity profiles as a net flow of bicycles. As a first advantage, this approach significantly simplifies the characterization of docking stations, avoiding other complex methods based on Markov chains and Poisson processes. Secondly, focusing on net flows rather than on occupancy, creates a solid basis for predicting of future states, given that it measures the evolution of the demand rather than its current state; note that a high occupancy with a zero net flow does not imply a potential lack of available docks in the near future.

This activity profile feeds the clustering algorithm HAC. We impose just a behavioural criterion to HAC and no geographical requirements. From the presented results, we observe that this geographical component emerges naturally from the HAC. Furthermore, it carries enhanced spatial information as it can group docking stations placed far away from each other but showing a clear spatial structure, for example, lying along the main South–North and East–West arterials of the city. This extra information could be considered to improve the design of rebalancing schemes.

As any other artificial intelligence tool, clustering requires a dataset with meaningful information. The data supplied by BiciMAD fulfils this requisite: trips performed by users are separated from those performed by staff (mainly due to rebalancing and repair operations); and they carry information about origin and destination, which avoids the construction of complex statistical models in order to estimate the interrelation between docking stations.

Models and algorithms that deal with behaviour are always difficult to validate as they lack of a clear-cut ground truth [25]. This usually leads to using qualitative analysis that show the consistency of the observed results. Following this approach to validation, we have provided evidence about the soundness of the proposed HAC-ULECA by means of: (i) comparative analysis of the weekly arrival and departure profiles of each resulting cluster; (ii) colour maps that visually show the distinct behaviour of the clusters; and (iii) geographical maps from which we can infer coherent mobility patterns within the city.

HAC-ULECA opens a new research line in the field of BSS modelling and optimization. It creates a clustering technique that does not impose proximity requirements to the algorithm, thus extending its applications beyond the mere reduction of rebalancing operations towards the extraction of knowledge about the mobility of the BSS and the city. In addition, it allows the adoption of edge-computing approaches, contributing to enhancing the functionalities of the IoSB architecture.

We will follow this new research line, using HAC-ULECA as the basis of our on-going research in the field of shared mobility in our group. First, we are working on extensions to the IoSB architecture which include incorporating the figure of the cluster coordinator. Data from the all the docking stations of a cluster will send information to one of them acting as the cluster coordinator. The cluster coordinator will register this information and compare it to the already known behavioural pattern within its cluster. This way, the cluster coordinator will be able to report to the higher layers in the IoSB architecture about deviations from the expected profile. Second, we will apply to BSSs prediction techniques already developed in the group in the field of mobility. This line of research will eventually lead us to propose new rebalancing schemes. Third, we will use the capability of HAC-ULECA of detecting docking stations with failures to develop a tool to anticipate deterioration events and trigger maintenance operations. Finally, following a complementary route to [26], we will analyse the performance of BSSs considering metrics that are not restricted to trips per bicycle and day but also how the existence of the BSS changes the city’s mobility patterns and reduces externalities such as pollution and comfort.
